# A Multilevel Approach to Explore the Wandering Mind and Its Connections with Mindfulness and Personality

**DOI:** 10.3390/bs11090125

**Published:** 2021-09-18

**Authors:** Damiano Cantone, Susanna Feruglio, Cristiano Crescentini, Sabrina Cinot, Alessio Matiz

**Affiliations:** 1 Department of Languages and Literatures, Communication, Education and Society, University of Udine, 33100 Udine, Italy; susanna.feruglio@uniud.it (S.F.); cristiano.crescentini@uniud.it (C.C.); alessio.matiz@uniud.it (A.M.); 2 Department of Psychology, Sapienza University of Rome, 00118 Rome, Italy; 3 Department of Medicine, University of Udine, 33100 Udine, Italy; sabrinacinot@gmail.com

**Keywords:** mind wandering, mindfulness, personality

## Abstract

We propose an innovative approach to study Mind Wandering (MW), and we present an application of this methodology to study the effects of a Mindfulness-Oriented Meditation (MOM) training. We assessed individuals’ MW through a free association task and an attentional task with thought-probes combined with a questionnaire for the phenomenological characteristic of each MW episode. We used the Temperament and Character Inventory to assess participants’ personality traits and their associations with measures of MW. Our study was limited by the course of the Covid-19 pandemic and only nine healthy young individuals completed the testing sessions, which were carried out before and after the MOM training. After MOM, participants showed fewer repetitive and self-relevant thoughts and indices of better performance in the attentional task; the linguistic analysis of participants’ free associations showed lower verbal productivity and a decrease in utterances that expressed anxiety/stress. Overall, we foresee that future studies could replicate our preliminary findings with larger samples and in a period without a global health emergency. This multilevel approach to the study of MW may allow researchers to gain a broader view of the phenomenon, considering its occurrence, qualitative characteristics, impact on cognitive tasks, malleability via mindfulness or other psychological interventions, and relations with personality traits.

## 1. Introduction

The last decades have seen increased research on spontaneous thoughts and mind wandering (MW), along with rising interest in understanding the cognitive and psychological processes involved in mindfulness-based meditation. These two lines of research appeared to have many common elements; however, the link between MW and Mindfulness Meditation (MM) remains an open question, also due to the difficulties in observing and measuring spontaneous thoughts. In this article we propose a new multilevel approach to study the impact of MM on the wandering mind of practitioners, which combines a first-person and a third-person perspective on MW. 

MW is a ubiquitous phenomenon that seems to profoundly characterize human experience, occupying a large part of peoples’ waking hours [1,2]. It can be defined as a type of self-generated thought independent from external stimuli that arises relatively freely and is only marginally bound to deliberate and automatic constraints (e.g., cognitive control and automatic mental habits [3]. Usually, MW is more frequent when people are in a resting state or are doing a low demanding activity [4]. It has been proposed that MW may be useful for carrying out some important psychological functions such as creative thinking and mental simulation [5]; nonetheless, in some circumstances it can have potentially negative consequences. For example, MW has been associated with increased errors and other performance impairments in activities that require focused attention, such as reading comprehension, attentional and memory tasks [6,7,8]. Neuroimaging studies have shown a prominent activation of the default mode network (DMN [9]; when individuals experience MW; this brain system comprises areas of the frontal and parietal lobes which have been repeatedly linked to self-generated cognition [3,10,11,12].

Most studies on MW focus on its occurrence and use first-person measures, such as experience sampling [4]; participants are probed about their current state of mind at random intervals (probe-caught MW), or they spontaneously report each time they become aware of having just had a MW episode (self-caught MW). These self-report measures of MW may be informative, but might also not be accurate due to the limitations of introspection and thus need to be triangulated with third-person measures, such as the observation of changes in brain activity, body indices and behavior. These measures mainly involve indirect indices of MW occurrence, e.g., altered attentional performance [13,14], neurophysiological changes in DMN activity [12,15], eye blink [16], etc. Research on spontaneous thoughts has advanced significantly thanks to the new neuroimaging techniques; however, the phenomenological characteristics of MW still has received little attention. The contents of any MW episode can be organized along different dimensions including emotional valence, temporal orientation, intensity, self-relevance, intentionality, etc. [17,18]. Some authors [13] tried to deepen the investigation of the functions and phenomenology of MW using a novel experience sampling method during the execution of an attentional task. They found that, in a sample of healthy university students, MW episodes mainly consisted in realistic and involuntary future-oriented thoughts about practical plans or concerns related to the present day. Interestingly, a subsequent study showed that some characteristics of MW appear to be stable over time and are related to personality traits [19]. More specifically, higher levels of self-directedness, i.e., the ability to regulate behaviors according to external demands and in line with personal motivations [20], were related to less MW and higher ratings of comfort during a resting-state period; conversely, the level of comfort was negatively associated with neuroticism-related personality traits [19], and neurotic individuals reported more MW episodes during the execution of cognitive tasks [21].

One practice through which people may train their ability to recognize MW episodes and better regulate them could be MM. Mindfulness has been defined as a kind of awareness intentionally directed towards present moment experience, characterized by an attitude of curiosity, openness and equanimity [22,23]. This state of mind can be trained through the practice of mindfulness-based meditation, which are a heterogeneous family of techniques, such as breath meditation, body scan, open monitoring, etc. The practice of MM has been linked to many positive outcomes in both clinical and healthy populations, e.g., enhanced attentional skills, better emotion regulation, reduced symptoms of anxiety, depression and stress [24].

Through MM, practitioners become familiar with the observation of ongoing spontaneous thoughts, while maintaining a non-judgmental and non-reactive attitude. Recently, some authors [25] have proposed that the practice of MM may influence the spontaneous wandering of the mind through different processes: increasing the meta-awareness of MW, enhancing attentional control skills, de-automatizing the emotional response to MW contents, and allowing practitioners to embrace an accepting attitude towards MW. Furthermore, a systematic review of the literature carried out by our research group (currently under review) has corroborated the hypothesis that MM has an influence on the wandering mind of naïve individuals, beginner and expert practitioners. More specifically, some studies have shown that mindfulness-based trainings (at least two weeks of training) reduced the occurrence of MW episodes and limited its negative effects on different cognitive tasks, such as attentional and memory tasks [26,27,28,29,30]. In addition, the comparison between long-term meditators and naïve individuals revealed some differences: meditators self-reported less MW and showed relatively lower activity in the DMN, during meditation and in a resting-state (e.g., [31,32,33]). 

In the present article, we propose an innovative approach to deepen the study of the effects of MM on the wandering mind. In accordance with the importance of triangulating different measures of MW, we combined a free associations task with an attentional task with thought-probes and a retrospective questionnaire on the contents and characteristics of MW episodes that occurred during the task [13,14]. The free associations task is inspired by the classical psychoanalysis method through which patients are invited to verbalize their own mental contents using introspection [34]. Similar protocols, like thinkingoutloud, were used in research to study cognitive processes such as problem solving [35]. More recently, this method has been re-proposed in the neuroscientific field to examine the description of the emotional states of individuals in situations of stress and psychodynamic conflict [36,37]. In the task proposed here, participants were asked to verbalize their mental thoughts while letting their mind wander spontaneously, reporting the content of their conscious experience moment by moment. All the speech produced by participants in this task was recorded without interfering with the spontaneity of thought flow and then analyzed with a linguistic analysis of speech, considering both the macro-linguistic level, the micro-linguistic level and the contents of the discourse [38]. Thus, through this methodology we can gain new insights into the incidence and the qualitative aspects of MW. The free associations and the attentional tasks were completed twice by a group of healthy young individuals before and after a MM training lasting eight weeks. Finally, we also aimed to explore the relationship between MW episodes, MM practice, and personality, as some personality traits appeared to be related to stable MW dimensions [19,21], and MM trainings have shown to positively affect individuals’ personality toward healthier profiles [39,40].

## 2. Experimental Objectives

The main goal of the experiment was to use the proposed approach to measure the impact of an eight-week Mindfulness Oriented Meditation (MOM) [41] training on participants’ MW. As mentioned in the Introduction, numerous studies have shown that MM can reduce the number of practitioners’ MW episodes; however, less is known about the impact of MM on the phenomenology of MW episodes. For this purpose, we adopted two different methods of evaluation of MW: a Sustained Attention to Response Task (SART) with thought-probes combined with a Thought Characteristics Questionnaire (TCQ), and a free association task. We therefore propose a multilevel approach to measure MW phenomena both quantitatively and qualitatively. A secondary, but significant goal was to relate the different measures of MW with participants’ personality traits. Thus, we used the Temperament and Character Inventory (TCI) to assess participants’ temperament and character. The same assessment was carried out before the beginning and after the end of the MOM training.

## 3. Methods and Materials 

### 3.1. Participants

The experiment involved a group of 35 healthy young adults, all medical students in their fourth year (11 men, 24 women). This experimental group was homogeneous in age (M = 23.0 ± 1.1; range: 22–26 years). The group was recruited on a voluntary basis from the participants of an eight-week MOM program specifically organized for medical students. Of the initial sample, nine subjects completed the experiment (3 men and 6 women; M age = 22.7 ± 0.7) (see below for more details). Informed consent was obtained from all participants. The procedures were approved by the local Ethics commission of the University of Udine and were in accordance with the Helsinki Declaration guidelines.

### 3.2. Procedure

Participants were tested before the beginning and after the end of the MOM training. The first assessment session was carried out from December 2019 until 9 January 2020. The week after, the first MOM meeting took place on 16 January 2020 (14:00–18:30). The second meeting followed on 28 January 2020 (14:00–18:30). Due to the evolution of the COVID-19 pandemic, after these two meetings it was no longer possible to proceed in person with the other two meetings of the course. The latter were conducted through pre-recorded video-lessons and meditations and sent to participants on 22 March 2020. Due to the lockdown imposed by the government to control the spread of the pandemic and the difficulty of organizing face-to-face meetings, the repetition of tests in the second testing session was extended over a period of five months from mid-May to mid-October 2020. Of the initial sample of 35 students, only nine completed the MOM training individually at home; self-reported to have meditated at home until the second test session; and took part in both the first and second assessment sessions. During these sessions, participants performed the SART with thought-probes, the TCQ and the free association task. Moreover, they were asked to complete the TCI online at home. Before the beginning of the SART the procedure was explained to each participant. They were told how to distinguish three types of thoughts that might have arisen during the task: (1) mind wandering episodes; (2) thoughts related to the task (e.g., “I should press the space bar faster”); and (3) thoughts triggered by environmental or physical stimuli (e.g., “it’s very cold in this room”). For the purpose of this study, they had been told that only the first kind of thoughts had to be reported. At the end of the SART, each participant was told to rate each episode of MW reported during the task according to an Italian translation of the TCQ items derived from Stawarczyk’s work [13,14]. Then, at the end of the TCQ questionnaire, each participant was interviewed by a researcher and invited to briefly describe the thoughts reported. Finally, the 20-min free association task was audio recorded and, at the end of the experiment, the free associations were transcribed and a linguistic analysis of the speech was conducted.

### 3.3. Temperament and Character Inventory 125 (TCI)

The Italian adaptation of the TCI-125 (validated by [42]) was used. This version is based on the original questionnaire developed by [20]. The TCI includes four temperamental and three character scales. The temperament reflects the way in which individuals accomplish a complex series of automatic reactions to environmental stimuli. People tend to respond in a similar way in similar situations. The four temperamental TCI dimensions are: (1) Novelty Seeking (NS), associated with exploratory activity in response to new environmental stimuli, impulsiveness, sudden mood swings and avoidance of frustration; (2) Harm Avoidance (HA), characterized by anticipatory worry, pessimistic attitude, and fear of uncertainty; (3) Reward Dependency (RD), describing high sensitivity to reward cues, especially verbal cues of social approval and social support; and (4) Persistence (P), related to the ability to persist in an activity despite fatigue or frustration. On the other side, the character reflects the result of a person’s development in learning concepts of self, intentional values and personal goals in a propositional or intuitive way. Character matures over time in a non-linear way and is defined at three levels: intrapersonal, interpersonal and transpersonal. Self-directedness (SD, intrapersonal level) denotes individuals’ ability to control their behavior to adapt to the environment and to achieve their goals. It is an index of maturity, responsibility, and reliability. Cooperativeness (C, interpersonal level) indicates attention to others, acceptance of others, willingness to cooperate, and the ability to be part of a community or a group. It expresses empathy, tolerance and supportive ability. Self-transcendence (ST, transpersonal level) refers to the ability to integrate one’s existence into a whole, to feel part of a larger reality (nature, universe, etc.). It is a sign of creativity, altruism and spirituality.

### 3.4. Sustained Attention to Response Task (SART) with Thought-Probes

The study of MW was conducted via the protocol developed by [13,14], namely through a computerized SART with thought-probes. The task took place in a quiet room, with the participant and the researcher present together. We used a 15′ laptop with an external keyboard. The participant sat in front of the screen (approximately 70 cm away) and the researcher sat next to the participant. Participants had to respond as quickly and carefully as possible by pressing the spacebar when a digit appeared on the screen (numbers 1–9), inhibiting their response in case of target stimulus onset (number 3). The digits were presented in sequences in random order one after the other at the center of the screen, with a presentation time of 500 ms and an inter-stimulus interval of 2000 ms. The sequences were 10, 14, 18, 22 or 26-digits long and each of them was repeated six times (for a total of 30 sequences). The sequence length selection order was random. 

Each sequence ended with a thought-probe: a question about having experienced episodes of MW during the sequence of digits just passed (“Have you had any thoughts, unrelated to the task you are performing, in the sequence of numbers just ended?”). If there were no episodes of MW in the recently ended sequence of numbers, participants had to press the letter N (“no”) and the task resumed. In case of episodes of MW, they had to press the letter S (“yes”) and were then asked to input a label (i.e., the thought’s “title”) that would allow them to recall that specific thought at the end of the whole task. Overall, the SART lasted approximately 25 min.

### 3.5. Thought Characteristics Questionnaire (TCQ)

This self-report questionnaire was originally derived from the Memory Characteristics Questionnaire created by [43], which was used to describe phenomenological characteristics of thoughts sampled in daily life (e.g., [44,45]). For each thought reported at the end of the number sequences included in the SART task, 10 characteristics were assessed. The first group of items were evaluated on a Likert scale ranging from 1 = “not at all” to 7 = “totally” and referred to: (tcq1) visual imagery; (tcq2) inner speech; (tcq3) the voluntary aspect of thought occurrence; (tcq4) how much the thought belonged to a structured sequence of thoughts, such as in problem solving; (tcq5) how much its content was realistic and plausible; (tcq6) how much its content was related to the participant’s personal experience; (tcq7) how much its content was important to the participant’s life; and (tcq8) how much its content was related to the participant’s personal goals. This was followed by two items assessing (tcq9) the recurrence of the specific thought in daily life (1 = “never occurs in daily life”, 7 = “occurs very often in daily life”), and (tcq10) its affective valence (0 = “very negative”, 4 neutral content 7 = “very positive”). Finally, the participants were asked to indicate (tcq11) the temporal dimension of their thoughts (past, future, neither). 

### 3.6. The Free Association Task

MW was also investigated through a new experimental approach, based on the free association method already used by Freud [34,37]. During the task, participants were invited to verbalize all their thoughts without attempting to control the contents of their conscious mind; the subsequent analysis of speech could help to identify possible underlying conscious/unconscious schemes of thoughts (see Supplementary Materials for details on the linguistic analysis). 

After the SART task, the participants were left alone in the experimental room. They lay in a comfortable position (lying down on a sunbed). After that, a voice recorded in a computer located in the same room asked participants to try to articulate all the thoughts that were going through their mind: “During this exercise you have to say out loud without any censorship what is on your mind, such as thoughts, images, memories, fantasies. In the exceptional case that there are thoughts or memories that you want to keep absolutely confidential, please say the sentence: “I do not say this thought”. Then go on with the assigned task, and try to say all the other thoughts aloud”. The task lasted 20 min. At predetermined intervals (every four min) the instructions were repeated. 

### 3.7. Mindfulness Oriented Meditation (MOM) Training 

The original MOM training [41,46,47] is an eight-week program with a 2-h group meeting every week and 30-min daily meditation practice at home. In its structure the program is similar to the Mindfulness-Based Stress Reduction course, the most common MM program developed by [48] In this study, the MOM training was slightly different: each group meeting lasted 4.5 h and brought together the activities of two 2-h meetings of the course as usually structured with a thirty-minute break halfway through. The meetings were originally scheduled every two weeks but, as described in the text above, the last two meetings were delivered together as recorded video-lessons. In total, the course lasted for 10 weeks. Each 2-h session was organized in three parts: a teaching on topics related to the practice of mindfulness (30 min), a guided meditation (30 min) and a final phase during which participants could share their experiences and pose questions to the instructor (this final phase was not present in the last two course blocks). The meditation practice, which was the same throughout the course, consisted of 10 min of paying attention to the breath (*anapanasati* meditation), 10 min paying attention to bodily sensations (body scan meditation) and 10 min paying attention to emotional and mental phenomena (*vipassana* meditation). These practices were adapted from the traditional Buddhist meditations as explained in the Satipatthāna discourse [49]. The same three meditative practices are part of other psychologically-oriented MM courses [48]. 

### 3.8. Statistical Analyses

Three main analyses were conducted: descriptive statistics, before vs. after MOM comparison of means, and correlation analysis.

Descriptive statistics were calculated for TCI scales, SART performance, TCQ dimensions, and linguistic and thematic indices of speech obtained from the free association task. The TCI scales have been described in Section 3 of Methods and Materials (NS, HA, RD, P, SD, C, ST); the SART performance was evaluated (during the whole task, in blocks where MW was reported and in blocks where no MW was reported, respectively) using the mean accuracy in responding to the target stimuli (ACC) and the mean reaction time to the non-targets (RT); the TCQ dimensions have been described in Section 5 of methods and Materials (tcq1-tcq10); the linguistic indices of speech were the number of Units and Utterances (as a measure of verbal productivity), the percentage of Units with Disfluencies (as a measure of verbal uncertainty), the percentage of Utterances with Cohesion Errors (as a measure of discourse elaboration) (for a detailed description of these indices see Supplementary Materials, see also [38] for more details), while the thematic index of speech was the percentage of Utterances in which participants told about their personal anxiety or stress (e.g., physical complaints, fears for the future, fatigue, worries about exams, ruminations about academic duties).

Before vs. after MOM comparison of means concerned the same measures described for the baseline analysis. For all measures, comparison of means was carried out with robust paired *t*-tests.

Correlational analysis was employed to examine the relationship between individuals’ TCI scores (averaged over the two assessments, i.e., before and after MOM) and post-pre MOM changes of the measures where significant differences were observed in the before vs. after MOM comparison of means. Overall, due to the exploratory nature of the present study, the conventional level of *p* < 0.05 was used as a statistical threshold of significance.

## 4. Results

### 4.1. Personality

The individual raw scores of the TCI scales were initially converted to z-scores by using the age- and gender-matched average scores of a large control sample of healthy individuals (see Table S3 in [50]). As summarized in Table 1, the participants’ average personality scores at baseline were in the normal range, being within 1 SD with respect to normative data. After the MOM training, these scores did not change significantly (for all, |t| < 1.7, *p* > 0.13), with the exception of the C scale (t = 4.0, *p* = 0.004), where a significant decrease was observed.

### 4.2. SART Performance

At baseline, participants’ mean accuracy in responding to target stimuli (ACC) included in the SART task was 64.9% and the mean reaction time to the non-targets (RT) was 390.3 ms (see Table 1). Participants reported a mean of 12.2 MW episodes across the 30 thought-probes of the SART task. In blocks where MW was reported, accuracy to the target stimuli was significantly lower than in blocks where no MW was reported (in blocks with MW, ACC = 56.8%, in blocks without MW, ACC = 71.9%; t(8) = −2.7, *p* = 0.03). In blocks where MW was reported, RT to the non-targets was similar to that of blocks where no MW was reported (in blocks with MW, RT = 390 ms, in blocks without MW, RT = 393 ms; t(8) = −0.3, *p* = 0.76).

After the MOM training, participants’ SART performance generally improved: the overall accuracy to the target stimuli increased (ACC = 69.1%), although not significantly (t(8) = −1.1, *p* = 0.30), and the overall RT to the non-targets significantly decreased (RT = 378 ms; t(8) = 2.4, *p* = 0.04). This decrease was reflected in the pre vs. post significant decrease of RT during blocks without reported MW (t(8) = 3.1, *p* = 0.02), while the pre vs. post decrease of RT during blocks with reported MW, as well as the increased accuracy during blocks with and without MW, were all not significant (for all, |t(8)| < 1.4, *p* > 0.21). Even the small increase in the number of reported MW episodes across the 30 thought-probes (+1.5) was not significant (t(8) = −0.7, *p* = 0.53).

### 4.3. Characteristics of Mind-Wandering Episodes

Before the MOM training, MW episodes occurring during completion of the SART task and evaluated via the TCQ, had, on average, the following characteristics (summarized in Table 1): they contained a moderate amount of visual imagery (M_tcq1 = 3.8, on the 1-to-7 Likert scale used for TCQ questions from tcq1 to tcq10) and a mild amount of inner speech (M_tcq2 = 3.1); they were mostly not intended (M_tcq3 = 2.4) and not part of a structured sequence of thoughts (M_tcq4 = 2.5); they were extremely realistic (M_q5 = 6.5) and their content appeared to be concrete and related to participants’ personal experience (M_tcq6 = 4.6); they were moderately important (M_tcq7 = 3.5) and slightly related to the participants’ personal goals (M_tcq8 = 2.9); they were slightly recurrent in participants’ daily life (M_tcq9 = 3.0) and their affective tone was neutral, despite showing a small negative bias (M_tcq10 = 3.9). Finally, about one in four (23.4%) of these thoughts was situated in the past, about one in four (24.1%) in the future, and the remaining 52.5% of thoughts were neither in the past nor in the future.

The characteristics of the MW episodes occurring during the SART task completed after the MOM training were significantly different in five TCQ dimensions compared to the MW episodes occurring before the MOM training. The thoughts that occurred after the MOM training appeared to be less realistic (M_tcq5 = 6.0, t(8) = 3.2, *p* = 0.012), concrete (M_tcq6 = 3.2, t(8) = 2.9, *p* = 0.02), important (M_tcq7 = 2.6, t(8) = 2.5, *p* = 0.04), related to the participants’ personal goals (M_tcq8 = 2.0, q(8) = 4.8, *p* = 0.001) and recurrent in the participants’ daily life (M_tcq9 = 2.3, t(8) = 2.5, *p* = 0.04) compared to those that occurred before the MOM training.

### 4.4. Free Association Task

During the 20-min free association task performed before the MOM training, participants used on average 1261.4 linguistic Units in 129.1 Utterances. Disfluencies were found in 3.9% of the Units pronounced, Cohesion Errors in 6.2% of the Utterances used and the topic of Anxiety/Stress in 19.9% of the total number of Utterances (see Table 1).

During the repetition of the task performed after the MOM training, participants used significantly less Units (M = 563.3, t(8) = 2.7, *p* = 0.03) in non-significantly less Utterances (M = 53.6, t(8) = 2.2, *p* = 0.055). The Percentages of Disfluencies (found in 3.2% of the Units) and of Cohesion Errors (found in 5.0% of the Utterances) did not change (t(8) = 1.2, *p* = 0.27; t(8) = 0.5, *p* = 0.61 respectively). The percentage of Utterances containing the topic of Anxiety/Stress decreased significantly to 7.5% (t(8) = 2.6, *p* = 0.03).

### 4.5. Correlations

Post- minus pre-MOM training significant changes in TCQ scores, in the indices of SART performance and in the linguistic measures of the free association task were correlated with TCI scores (averaged over the two assessments, one before and one after the MOM training). As summarized in Table 2, changes in tcq7, tcq8 and tcq9 dimensions of MW episodes during SART were inversely related to ST levels (all r < −0.69, *p* < 0.04), while changes in RT during SART were directly related to ST levels (r = 0.80, *p* = 0.01). Changes in tcq8 scores were also inversely related to RD levels (r = 0.76, *p* = 0.02). Finally, changes in the percentage of Utterances with Anxiety/Stress during the Free associations task were inversely related to NS levels (r = 0.73, *p* = 0.03). In other words, greater reductions (from before to after the MOM training) in the importance, goal-orientedness and repetitiveness of MW thoughts during the SART task were associated with a higher level of transpersonal wellbeing (ST); while a greater improvement in SART RT performance was associated with a lower level of transpersonal wellbeing (ST). Moreover, the decrease in goal-orientedness of MW thoughts was also related to a higher level of sensitivity to social approval/support and sentiment (RD). Finally, during the FA task, greater reductions in Utterances with Anxiety/Stress elements were associated with a stronger tendency towards exploratory activity (NS).

## 5. Discussion

In the present article we proposed an innovative multilevel approach to study spontaneous thoughts and MW, and we presented a pilot study for a possible application of this methodology to study the effects of a mindfulness-based training course on individuals’ MW. Participants were assessed through a personality test (TCI), a computer attention test (SART) with thought-probes for detecting MW, a questionnaire for the phenomenological characteristic of each MW episode (TCQ), and a free association task. Given that MW has recently become a prominent field of research within cognitive neuroscience and psychology, this study aims to offer some suggestions to analyze this complex construct more broadly and deeply.

In particular, the linguistic analysis of free association allowed us to examine MW, not simply considering them in relation to an ongoing task, as in the SART protocol [13,14] but as a member of the family of spontaneous-thought processes—a family that also includes creative thoughts and dreaming [3]. Another novelty element of the present study consists in the possibility of comparing measurements carried out on different levels that integrate a first-person perspective (assessed through self-reports and free associations) with a third-person perspective on MW (assessed through the attentional task). This allowed us to gain a broader view of the MW phenomenon, considering its occurrence, qualitative characteristics, impact on cognitive tasks, and its relations with personality traits.

Although only a small number of healthy young individuals (nine people) completed the entire experiment, thereby limiting the possibility to draw general conclusions, a number of relevant methodological and theoretical considerations can be advanced. A certain degree of consistency is present across the results obtained using the different methods employed in the study. In particular, after the MOM training, participants showed a trend toward an overall increased accuracy in the SART task and a significant reduction in RT to non-target stimuli. A reduction in the degree of realism, concreteness, goal-orientedness, importance for personal life and repetitiveness of thoughts during MW episodes was also reported during the SART task. These results may be consistent with what emerged from the linguistic analysis of free association, which revealed a lower level of productivity and a marked decrease in the number of utterances that expressed anxiety and/or stress after the MOM training.

After the MOM training, we thus found fewer repetitive and self-relevant thoughts and indices of better performance in the SART task. If we take into consideration the objectives that MM proposes, such as observing, acting with awareness, non-judging, and non-reacting to internal experience, our findings seem to be consistent with the expected results. For example, MOM led to reduced repetitiveness of thoughts during the sustained attention task. This may have made such thoughts less intrusive and distracting during task execution, with the possible effect of reducing the need to react to them, thus improving attention and present-moment awareness (namely better performance in the SART). As mentioned above, the analysis of the TCQ responses reflecting the contents of MW episodes also showed a marked decrease in self-focus, as well as a reduction in realism and concreteness scores. Overall, this may indicate that the participants were more able to decenter (i.e. to distance themselves), during SART execution, from worries and thoughts derived from their daily life. In general, these results are in line with those obtained from a large series of studies on the impact of various forms of meditation on MW [51,52,53,54].

Furthermore, the variations in the values of the linguistic analysis of free association points in the same direction. A free association task could encourage MW, because participants are invited to become aware of the thoughts that pass through their minds and verbalize them, without any other specific request or aim. The outcomes measured in this task after the MOM training showed that participants used less words in general and were less likely to produce utterances with anxiety/stress contents. Verbal productivity might be interpreted as a reflection of a defensive process [55]. In fact, hyper-productivity can be an attempt to avoid or deny painful material. The reduction of verbal productivity in our sample may reflect a decrease in the participants’ need to protect themselves from eventually facing psychologically painful mental thoughts. Such a possibility is in line with the teachings of MM that propose to maintain an accepting and non-reactive attitude towards all present-moment experiences [56].

Finally, with regard to the correlations between participants’ personality traits and the changing in MW characteristics from before to after the MOM training, we found that the character dimension of self-transcendence was positively associated with the reduction in the importance, goal-orientedness and repetitiveness of MW episodes. It is possible that MM helped practitioners to widen their perspective about themselves, embracing a point of view less focused on their personal needs and plans. These findings would be in line with the process of change in the perspective on the self, including the self-transcend dimension, that some authors identified as a key component of mindfulness meditation practices [57,58,59]. Having said this, it is remarkable to consider that the only pre-post MOM change concerning personality involved a decrease in the cooperativeness of the participants following the MOM training, a result that stands in contrast with those of other studies (e.g., [39]). A possible explanation for this result is to postulate a negative involvement of social and physical distancing imposed by the authorities to contain the progress of the Covid-19 epidemic, which concerned the period in which the second test session was carried out. In general, this possibility leads us to be cautious in interpreting this result as concerns cooperativeness.

In conclusion, our study has important limitations, mostly bounded by the difficulty in carrying out the experiment as planned during the first wave of the Covid-19 pandemic. We hope to encourage future studies to replicate our preliminary findings in larger samples and during a period where there is no health emergency. A multilevel measurement of the impact of MM on the wandering mind of participants may contribute to showing how mindfulness practices could not only quantitatively reduce the number of MW episodes, but also influence their phenomenology. In other words, MM may be effective not only in reducing episodes of MW and in improving sustained attention task performance, but also in reducing the percentage of negatively self-focused and stress-related thoughts. This is one direction in which future studies could work experimentally, considering the wide spectrum of elements that can emerge from a multilevel approach to the study of MW and spontaneous thoughts.

## Figures and Tables

**Table 1:** Descriptive statistics of data obtained before and after the MOM training and results of paired t tests for each pair of measurements.

		Before MOM	After MOM	*t* Test	
				Statistic	*p* Value
**TCI**	**NS**	−0.3 ± 0.8	−0.4 + 1.2	0.2	0.816
	**HA**	0.7 ± 1.2	0.1 + 1.1	1.7	0.130
	**RD**	−0.1 ± 1.1	−0.3 + 1.1	1.0	0.342
	**P**	−0.2 ± 1.4	0.0 + 1.2	−1.4	0.191
	**SD**	−1.0 ± 1.1	−0.5 + 1.2	−1.5	0.174
	**C**	−0.1 ± 1.1	−0.7 + 1.2	4.0	0.004 **
	**ST**	0.7 ± 1.1	1.0 + 1.3	−1.3	0.245
**SART**	**ACC**	64.9% ± 9.5%	69.1% + 19.0%	−1.1	0.299
	**RT**	390.3 ± 21.8	378.4 + 25.2	2.4	0.040 *
	**numMW**	12.2 ± 5.5	13.7 + 5.3	−0.7	0.532
	**ACC_MW**	56.8% ± 13.5%	59.4% + 24.3%	−0.4	0.685
	**ACC_nMW**	71.9% ± 11.4%	76.8% + 20.4%	−1.1	0.286
	**RT_MW**	390.3 ± 23.6	381.2 + 26.4	1.4	0.209
	**RT_nMW**	392.8 ± 25.7	377.3 + 24.4	3.1	0.015 *
**TCQ**	**tcq1 (visual)**	3.8 ± 1.7	3.0 + 1.2	1.7	0.125
	**tcq2 (speech)**	3.1 ± 1.8	2.6 + 1.6	1.4	0.203
	**tcq3 (intended)**	2.4 ± 1.2	2.2 + 1.4	0.6	0.589
	**tcq4 (structured)**	2.5 ± 1.4	2.3 + 1.1	0.7	0.528
	**tcq5 (realistic)**	6.5 ± 0.5	6.0 + 0.6	3.2	0.012 *
	**tcq6 (concrete)**	4.6 ± 1.6	3.2 + 1.4	2.9	0.020 *
	**tcq7 (important)**	3.5 ± 1.4	2.6 + 1.0	2.5	0.039 *
	**tcq8 (goals)**	2.9 ± 1.1	2.0 + 0.7	4.8	0.001 **
	**tcq9 (repetitive)**	3.0 ± 1.0	2.3 + 0.7	2.5	0.035 *
	**tcq10 (affective)**	3.9 ± 0.4	4.1 + 0.3	−1.4	0.193
	**tcq11_%past**	23.4% ± 20.9%	19.6% + 16.7%	0.6	0.568
	**tcq11_%future**	24.1% ± 19.6%	18.4% + 8.9%	0.8	0.455
	**tcq11_%nPast-nFuture**	52.5% ± 23.8%	62.1% + 18.0%	−1.1	0.295
**FA**	**Units**	1261.4 ± 866.6	563.3 ± 535.1	2.7	0.026 *
	**Utterances**	129.1 ±94.8	53.6 ± 47.3	2.2	0.055
	**% Disfluencies**	3.9% ± 1.1%	3.2% ± 2.1%	1.2	0.269
	**% Cohesion Errors**	6.2% ± 5.4%	5.0% ± 4.0%	0.5	0.609
	**% Anxety/Stress**	19.9% ± 10.5%	7.5% ± 8.3%	2.6	0.031 *

Descriptive statistics are expressed as mean value ± standard deviation. Abbreviations: MOM = Mindfulness-Oriented Meditation; TCI = Temperament and Character Inventory, NS = Novelty Seeking, HA = Harm Avoidance, RD = Reward Dependence, P = Persistence, SD = Self-Directedness, C = Cooperativeness, ST = Self-Transcendence; SART = Sustained Attention Response Task, ACC = Accuracy, RT = Reaction Rime, numMW = number of Mind-Wandering episodes, ACC_MW = Accuracy in SART blocks with reported Mind-Wandering, ACC_nMW = Accuracy in SART blocks with no reported Mind-Wandering, RT_MW = Reaction Time in blocks with reported Mind-Wandering, RT_nMW = Reaction Time in blocks with no reported Mind-Wandering; TCQ = Thoughts Characteristics Questionnaire; FA = Free associations; * = *p* < 0.05; ** = *p* < 0.01.

**Table 2:** Correlation coefficients between individuals’ TCI scores (averaged over the two assessments, i.e., before and after MOM) and post-pre MOM changes of the measures where significant differences were observed in the before vs. after MOM comparison of the means.

		TCI						
		NS	RD	P	HA	SD	C	ST
**TCQ**	**delta tcq5**	0.39	0.33	−0.13	−0.15	−0.54	0.08	−0.61
	**delta tcq6**	0.31	−0.47	−0.55	−0.27	−0.32	−0.62	−0.23
	**delta tcq7**	0.01	−0.27	−0.42	0.10	−0.53	−0.32	−0.77 *
	**delta tcq8**	0.05	−0.76 *	−0.57	0.05	−0.42	−0.64	−0.69 *
	**delta tcq9**	−0.16	−0.39	−0.32	0.03	−0.32	−0.29	−0.73 *
**SART**	**delta RT**	−0.16	0.45	0.23	−0.02	0.33	0.47	0.80 *
**FA**	**delta Units**	−0.27	−0.05	0.50	−0.45	0.57	0.05	0.58
	**delta % Anxiety/Stress**	−0.73 *	−0.22	−0.10	0.13	0.35	0.30	0.38

Abbreviations as in Table 1. Asterisks indicate *p* < 0.05.

## Data Availability

The data that support the findings of this study are available from the corresponding author, upon request.

## Supplementary Materials

The following are available online at https://www.mdpi.com/article/10.3390/bs11090125/s1, Linguistic analysis of Free association Task. References [38,60,61,62,63].
