# Supplementary material for: A Multilevel Approach to Explore the Wandering Mind and Its Connections with Mindfulness and Personality
Source: Behav Sci (Basel). 2021 Sep 18;11(9):125. doi: 10.3390/bs11090125 (PMC8469085; doi:10.3390/bs11090125)
Supplement: Supplementary file 1 [file behavsci-11-00125-s001.zip › behavsci-1340251-supplementary.pdf]

Linguistic Analysis

(based on Fabbro et al. [38])

For the aims of the present research, the following linguistic indices were taken into account: (1) the number of Units and the number of Utterances as a measure of verbal productivity, (2) the percentage of Units with Disfluencies as a measure of linguistic uncertainty, (3) the percentage of Utterances with Cohesion Errors as a measure of discourse elaboration. The linguist indices were obtained as follows.

1) Units are words or incomplete words: clitics have been counted as separate Units, while Italian articulated prepositions as consisting of two Units. Utterances (separated with / in the following examples) are obtained by segmenting the text with the following criteria ([60] (p. 201); Loban [61]):
    a. an utterance is "a self-contained segment of speech that stands on its own and conveys its own independent meaning" [62];
    b. a sentence can be an utterance, but some utterances are compound sentences, while some are incomplete sentences (e.g., a false start);
    c. a segment shorter than a sentence that does not form a syntactic unit with what follows is considered as one utterance;
    d. intrasentential pauses do not determine the interruption of an utterance (a sentence with an intrasentential pause is counted as one utterance; e.g., "/Her name is ... Paula and her surname Sangoi/");
    e. repetitions do not determine the interruption of an utterance (a sentence with an intrasentential pause is counted as one utterance; e.g.: "/There is a ... *a* tree/");
    f. interruptions (of words or sentences) with revisions of words do not determine the interruption of an utterance (a sentence with an interruption with revision of words is counted as one utterance; e.g.: "/The cat is playing without *with* the dog/"; e.g., "/There are two pens *two pencils*/"), but when interruptions concern the structure of the sentence, or an utterance is interrupted and left incomplete, the end of the utterance is counted (e.g.: "/*I know that the woman*/ I know that the car of the police/"; e.g. "/*The nest fell down with*/ eggs were broken/");
    g. filler words (e.g., "yes", "okay", "right", "you know") do not determine the end of an utterance (e.g., "/The man... okay... was very confused/");
    h. parenthetical utterances are considered independently from the main utterance (a parenthetical utterance is counted as one utterance; e.g.: "/the brother /*I don't know who he is*/ gives a gift to his sister/" contains two utterances; e.g., "/two guys /*I think brother and sister*/ are walking down the street/" contains two utterances).
2) Disfluencies include revisions, repetitions, fragments of words and filler words [61]. The percentage of Units with Disfluencies is calculated as follows: [(revised words + repetitions + incomplete words + filler words) / units] * 100.
3) Cohesion Errors concern the structural relationships between contiguous Utterances (Marini et al., [63]). We have considered Cohesion Errors as the incorrect use of cohesive functors (e.g., function words, anaphoric pronouns, errors in gender or number agreement across Utterances) and the abrupt interruption of an Utterance followed by a change of subject. The percentage of Cohesion Errors was calculated as follows: [(incorrect uses of cohesive functors + abrupt interruptions of an Utterance) / Utterances] *100.
